# Progress in State Medicine

**Published:** 1897-01-30

**Authors:** 


### Progress in State Medicine.

**Phthisis.**—Moore[1] draws attention to the fact that 12·3 per cent. of the deaths in England and Wales, 13·8 per cent. of the deaths in Scotland, and 14·9 per cent. of the deaths in Ireland are attributable to phthisis. The disease is undoubtedly infectious, and capable of being spread by the excretions and discharges of men and animals, by milk and by flesh. Amongst preventative measures, the most important are: Inspection of food, improved housing, and the provision of sanatoria for early cases; whilst those in a more advanced stage are treated in special hospital wards, or homes for the dying. Recent returns from Germany show the mortality from phthisis to be about 107 per 1,000 deaths, so that the high rate is in no way peculiar to this country. In England it has not yet been found advisable to add phthisis to the list of notifiable diseases, nor is it possible to treat any large proportion of the cases in hospital even if such a course were desirable. Sanitary authorities may, however, issue for general distribution leaflets of practical directions on the prevention of phthisis, and such a course has recently been followed in Glasgow. A more elaborate circular treating of the disposal of sputum, the disinfection, cleaning, and renovation of rooms occupied by tubercular patients, has been issued by the Sanitary Authority of New York.[2]

Niven, lecturing on the milk supply of Manchester, stated that 20 per cent. of cows were tubercular, and that amongst the stall-fed cattle of cities the figure was probably higher. He advocates the adoption of the Copenhagen system. Under this arrangement a large body of farmers send their milk to a central distributing depôt. The cows are carefully fed, cleaned, and groomed, and periodically inspected by veterinary experts. The milk drawn into ice is immediately despatched to the central station, where it is filtered through gravel, and finally delivered in sealed cans. Milk from diseased cows is destroyed, the owner being compensated.

Sydney Marsden[3] found about 20 per cent. of the cattle landed at Birkenhead to be tuberculous. He suggests that whenever the carcase of a beast thus diseased is seized, the cost should be equally divided, instead of falling upon one person as at present. In this way the farmer or importer would pay one-third, the butcher a third, and the sanitary authority a third. All parties would then be equally interested in detecting diseased meat.

With regard to the treatment of phthisical persons in hospitals, it should be noted that the committee recently appointed by the Paris municipal authorities to report upon the contagion of tuberculosis in general hospitals, has recommended that such patients shall in all cases be treated in separate wards or special hospitals. In America attempts have been made to show that such institutions may become centres of infection.

Knopf,[4] however, maintains that sanatoria for phthisis do not give rise to infection in their immediate neighbourhood, nor do patients suffer, if proper precautions be taken as regards the sputum.

**Disinfection of Rooms.**—The air of infected rooms can, by free ventilation, be changed and rendered innocuous. The ultimate aim, therefore, of all processes of disinfecting premises is to obtain a sufficient deposit of antiseptic material on the surfaces of walls, floor, and ceiling. Considerable doubt has of recent years been thrown upon the efficacy of sulphurous anhydride in performing this work.

Kenwood,[5] experimenting with diphtheria bacilli in a room of about 2,000 cubic feet capacity, found that if $SO_2$ were present to the extent of $\frac{1}{2}$ per cent., the microbes were killed after an exposure of from six to eight hours, and that growth was inhibited if the gas were present in the strength of $\frac{1}{4}$ per cent. No watery vapour was added, and the result was the same whether the gas was generated from cylinders containing it in the liquid state or from burning sulphur. From these results he is of opinion that the present methods fulfil all practical requirements. No test was made with more resistant and spore-bearing varieties of bacilli. Pfuhl[6] tested the value of formaldehyde vapour, generated from a Krell's lamp. After an exposure of from 18-24 hours, the more resistant and spore-bearing organisms were not destroyed, and he therefore judges the gas to be unsuitable for this purpose. Formalin (40 per cent. formaldehyde) he found to possess strong bactericidal powers, a 1 per cent. solution killing pure cultures of cholera and diphtheria bacilli in an hour. It is best employed, as recommended by England,[7] in a 2 per cent. solution to spray the walls and contents of the room, which is then closed for twenty-four hours. About 60-70 c.c. per square metre of surface are used. Klein[8] finds that sodium hypochlorite in solution of 1 in 200 destroys non-sporing microbes in five minutes, and even sporing organisms, such as anthrax, are disinfected in an hour and a-half. Tested on fresh sewage, a solution of 1 in 100 devitalises non-sporing forms in ten minutes. Such solutions would be valuable in domestic disinfection. There can be no doubt as to the superiority of spraying over fumigation. Even if we take into account the period which must elapse before the room be dry, the former process is the most economical of time. Spraying ensures more certainly the cleanliness of surfaces, but it is more easily scamped by negligent workmen than is our present process of fumigation.

**Water Supply.**—The report to the London County Council (Dibden) on the London Water Supply has given rise to much controversy. The analyses of Drs. Klein, Dupré, and Stevenson point to the existence of serious pollution, the result of defective filtration. The amount of suspended matter in many of the samples was excessive, and low forms of organic life, such as rotifera and protozoa, were detected. Not only was the number of bacteria excessive, but Dr. Klein succeeded in demonstrating the presence of B. Coli, pointing unmistakably to sewage contamination. These results, differing so widely from the official returns, were obtained by incubating the samples at a higher temperature, and for a longer period, than has been customary. It will be remembered that in 1894 the medical officer to the London County Council attributed the unusual prevalence of enteric fever at that time to a polluted water supply, and in the light of the present investigation this does not seem unlikely. The possibility of such an occurrence has recently been demonstrated at Chicago. The diagram[9] showing the relation between the sanitary character of the water supply and the deaths from enteric fever is very instructive, the increase and decrease of the degree of pollution being reflected in the rise and fall of the death line. Outbreaks or extensions of disease directly or indirectly dependent upon polluted water supplies are of frequent occurrence in our villages, where, in consequence of the public supply being absent, deficient, or difficult of access, water is taken from polluted sources such as ditches or surface wells.

Thresh[10] urges that in all cases villages should have a public water supply, either by pipe or from a deep well. It is always preferable to tap springs rather than to take the supply from streams. Ten to fifteen gallons per head per day is generally sufficient. The cost of works is borne by the whole village, whilst the expense of maintenance and pumping is met by a special rate levied on the persons benefited. The New York Board of Health, in order to facilitate correct diagnosis in doubtful cases of enteric fever, has undertaken to test bacteriologically the blood of such cases.[11] The test employed is founded on the observation that the blood of persons suffering from enteric fever when mixed with active cultures of the typhoid bacillus has the power of arresting the active movement of these organisms, and of producing peculiar and characteristic clumping of the bacilli. It is claimed that this property of the blood is peculiar to the special disease.

[1] Brit. Med. Journ., Oct. 10, 1896. [2] Sci. Bull. No. 12, New York. [3] San. Rec., Oct. 9, 1896. [4] Med. Rec., Oct. 3, 1896. [5] Brit. Med. Asso. Meeting at Carlisle, 1896. [6] Zeitschr. f. Hygiene xxii. [7] Om Formaldehyde, Stockholm, 1895. [8] Lancet, Nov. 28, 1895. [9] Journ. Am. Med. Asso., Oct., 1896. [10] Newcastle Cong. San. Inst., 1896. [11] Med. News, New York, Nov. 14, 1896.

